# Non-cognate translation priming in masked priming lexical decision experiments: A meta-analysis

**DOI:** 10.3758/s13423-016-1151-1

**Published:** 2016-09-09

**Authors:** Yun Wen, Walter J. B. van Heuven

**Affiliations:** School of Psychology, University of Nottingham, University Park, Nottingham, NG7 2RD UK

**Keywords:** Meta-analysis, Non-cognate masked translation priming, Bilingualism

## Abstract

The masked translation priming paradigm has been widely used in the last 25 years to investigate word processing in bilinguals. Motivated by studies reporting mixed findings, in particular for second language (L2) to first language (L1) translation priming, we conducted, for the first time in the literature, a meta-analysis of 64 masked priming lexical decision experiments across 24 studies to assess the effect sizes of L1–L2 and L2–L1 non-cognate translation priming effects in bilinguals. Our meta-analysis also investigated the influence of potential moderators of translation priming effects. The results provided clear evidence of significant translation priming effects for both directions, with L1–L2 translation priming significantly larger than L2–L1 translation priming (i.e., effect size of 0.86 vs. 0.31). The analyses also revealed that L1–L2 translation effect sizes were moderated by the interval between prime and target (ISI), whereas L2–L1 translation effect sizes were modulated by the number of items per cell. Theoretical and methodological implications of this meta-analysis are discussed and recommendations for future studies are provided.

The online version of this article (doi:10.3758/s13423-016-1151-1) contains supplementary material, which is available to authorized users.

## Introduction

A central issue addressed in bilingual psycholinguistic research is how words of two languages are represented and accessed. To investigate this fundamental issue with respect to visual word recognition, numerous studies have utilized the masked translation priming paradigm with a lexical decision task. Since the first translation priming effects were reported from the first (L1) to the second language (L2) in de Groot and Nas’s (1991) seminal study, follow-up studies have used the non-cognate masked translation priming paradigm to investigate both L1–L2 and L2–L1 priming in unbalanced bilinguals. These early studies have consistently reported significant L1–L2 translation priming effects, whereas they failed to find L2–L1 translation priming effects (e.g., Finkbeiner, Forster, Nicol, & Nakamura, 2004; Gollan, Forster, & Frost, 1997; Jiang, 1999; Kim & Davis, 2003).^1^

To explain this translation priming asymmetry found in early studies, two theoretical frameworks have been proposed in the literature. According to the episodic L2 hypothesis (Jiang & Forster, 2001; Witzel & Forster, 2012), L1 words are represented in lexical memory whereas L2 words are represented in episodic memory. This predicts that it is impossible for L2 primes to impact L1 targets. In contrast, the Sense Model (Finkbeiner et al., 2004) proposes that translation priming asymmetry is attributed to a representational asymmetry between L1 and L2 words, namely, L1 words are associated with more semantic senses than L2 words. As a consequence, semantic senses activated by L2 primes are insufficient to facilitate the recognition of L1 targets, whereas L1 primes can facilitate L2 targets. Both of these theoretical accounts predict that there is no L2–L1 translation priming. However, these models cannot account for some of the recent studies that found significant translation priming effects in both directions (e.g., Greek–English: Dimitropoulou, Duñabeitia, & Carreiras, 2011; Dutch–English: Duyck & Warlop, 2009; Japanese–English: Nakayama, Ida, & Lupker, 2016). In light of these recent findings, Schoonbaert, Duyck, Brysbaert, and Hartsuiker (2009) proposed a refined Distributed Representation Model (DRM), which was based on the distributed model of de Groot (1992). According to the DRM, L1 words are connected to more semantic nodes than L2 words, so L1 primes activate a larger proportion of semantic nodes of L2 targets than vice versa. Critically, the assumption of the DRM is that L1–L2 and L2–L1 translation priming are quantitatively rather than qualitatively different as implied in other models (Jiang & Forster, 2001; Kroll & Stewart, 1994; Witzel & Forster, 2012). Thus, the model predicts significant L2–L1 translation priming effects which are smaller than L1–L2 translation priming effects.

Several studies have provided narrative reviews of the asymmetric translation priming effects reported with unbalanced bilinguals in the literature (e.g., Altarriba & Basnight-Brown, 2007; Dimitropoulou et al., 2011; Nakayama et al., 2016; Xia & Andrews, 2015). As summarized in Dimitropoulou et al. (2011), only 8 out of 21 experiments reported significant L2–L1 translation priming (mean priming effect: 9 ms, ranging from –6 to 26 ms). Although robust L1–L2 translation priming was reported in all experiments (cf. Davis et al., 2010), the priming effects varied greatly from 16 to 100 ms (mean: 41 ms). A range of possible factors modulating the translation priming effects have been discussed in the narrative reviews, such as factors related to statistical power (i.e., number of participants), the prime-target presentation procedure (i.e., prime duration, inter-stimulus interval, stimulus onset asymmetry), the stimuli (i.e., number of experimental items, the languages involved are the same or different scripts) and general processing speed (i.e., response speed of participants). Because existing empirical studies differ dramatically in terms of all these factors and no studies in the literature have so far considered some or all of these potential moderators systematically, the tentative conclusions of narrative reviews remain inconclusive. Importantly, these insightful reviews have so far focused mainly on the magnitude of the priming effects (in milliseconds), which are unstandardized estimates of the effect sizes and thus cannot be compared across studies. Surprisingly, there are no meta-analytic reviews conducted in the literature, so far as we are aware, that quantitatively assessed the standardized effect sizes of L1–L2 and L2–L1 translation priming and the impact of potential experimental moderators on translation priming effects.

To fill this important gap in the literature, we present here a meta-analysis that investigated masked translation priming effects of non-cognates word pairs in lexical decision tasks. A meta-analysis uses standardized effect sizes and their variance observed in studies and tests statistically whether the overall effect size provides evidence of the experimental effect (Borenstein, Hedges, Higgins, & Rothstein, 2009). Another unique advantage of a meta-analysis is that potential moderators can be tested statistically. These moderators may explain inconsistent findings in experiments reported in the literature. Therefore, the aim of the meta-analysis in the present study was twofold. First, the primary goal was to determine the overall effect size of L1–L2 and L2–L1 translation priming and to statistically compare the effects sizes between the two translation directions. The second aim was to statistically test whether effect sizes of translation priming are influenced by moderators previously suggested in the literature. The following seven potential moderators were considered: the number of participants, the prime duration, the SOA (Stimuli Onset Asynchrony, i.e., the interval between the onset of prime and the onset of target), the ISI (Inter-Stimulus Interval, i.e., interval between the offset of prime and the onset of target), script type, number of items per cell and response speed.

## Method

### Literature search and study selection

A literature search was conducted using “masked translation priming” as the search string in PsycINFO, Web of Science and PubMed (up to 31 March 2016) to identify possible studies to be included in the meta-analysis. To find additional studies, recent studies and reviews of masked translation priming were consulted (Dimitropoulou et al., 2011; Duñabeitia, Perea, & Carreiras, 2010; Nakayama et al., 2016; Schoonbaert et al., 2009; Xia & Andrews, 2015). The following criteria were used to select the final set of studies and experiments for the meta-analysis: (1) prime duration ≤ 100 ms, (2) primes were masked, (3) a lexical decision task was used, (4) prime-target pairs were non-cognates, (5) L1/L2 of bilinguals were clearly specified, and (6) either the *F* or *t* value of the translation priming effect was reported. Using these selection criteria, we found 24 published articles. For the L1–L2 translation priming direction, 31 experimental observations were extracted from 20 studies. For the L2–L1 translation priming, 33 experimental observations were extracted from 18 studies. A detailed description of these studies is provided in the Supplementary Material.

### Meta-analysis

#### Effect sizes

The effect sizes (d) were calculated using *t* values or *F* values, the number of participants (*n*) and the formula proposed by Rosenthal (1991): $\mathrm{d}=\frac{t}{\sqrt{n\ }}$ or $\mathrm{d} = \sqrt{\frac{F\ }{n}}$. Previous meta-analyses (e.g., Van den Bussche, Van den Noortgate, & Reynvoet, 2009) have also used this formula to estimate effect sizes in within-subject experiments. In line with other meta-analyses for masked priming effects in monolingual studies (Lucas, 2000; Van den Bussche et al., 2009), *t* values or *F* values were taken from the subject analyses. To indicate the direction of the priming effects, the effect size was specified as positive or negative based on the means of the translation priming and unrelated (control) conditions. Thus, a positive effect size indicates a facilitatory translation priming effect. Sampling variance of the effect sizes was calculated using the formula provided by Morris and DeShon (2002):

$$ Sampling\  variance=\left(\frac{1}{n}\right)\left(\frac{n-1}{n-3}\right)\left(1+n{d}^2\right)-\frac{d^2}{{\left[c\left(n-1\right)\right]}^2} $$

in which d is the effect size, n is the number of participants and *c*(*n* − 1) is defined as (Hedges, 1981, 1982):

$$ c\left(n-1\right)=1 - \frac{3}{4\left(n-1\right)-1}\ . $$

#### Moderator coding

The seven factors mentioned in the introduction were included as moderators in the present meta-analysis. Six factors were included as continuous moderators: the number of participants, number of items per cell, the prime duration (in ms), the ISI (in ms), the SOA (in ms) and overall response speed as measured by the mean reaction time in the unrelated (control) condition (in ms). Script type was coded categorically, as either as same-script languages (e.g., Dutch and English) or different-script languages (e.g., Chinese and English).

#### Data analyses

The meta-analysis was conducted using the metafor package (Viechtbauer, 2010) in R v.3.2.4 (R Core Team, 2016). For both translation directions, a random-effects model without any moderators was first conducted to estimate the effect sizes of L1–L2 and L2–L1 translation priming. A *z* test was conducted to compare the overall effect sizes of the two translation directions (Borenstein et al., 2009). A significant *z* test would suggest that the effect sizes of the two translation directions are different and separate analyses are warranted. Next, for both translation directions, *Q* tests of variance were conducted to investigate the heterogeneity of the observed effect sizes. A significant *Q* test would indicate that the observed effect sizes are heterogeneous, and that potential moderators are likely to exist. To investigate the influence of the potential moderators, we used a meta-regression approach similar to Van den Bussche et al. (2009). First, each of the seven moderators were separately entered into a random-effects model. Next, when more than one of the moderators was significant, we included these significant moderators in the initial model of the meta-regression. In order to address the issue of the collinearity between moderators, we orthogonalised moderators that significantly correlated by fitting a linear model to obtain the residuals (see, for example, Siyanova-Chanturia, Conklin, & van Heuven, 2011, for a similar approach). The residuals of the model were then included in the meta-regression. Finally, a backward model selection procedure was used in which non-significant moderators were step-by-step eliminated from the model.

## Results

**Fig. 1:**
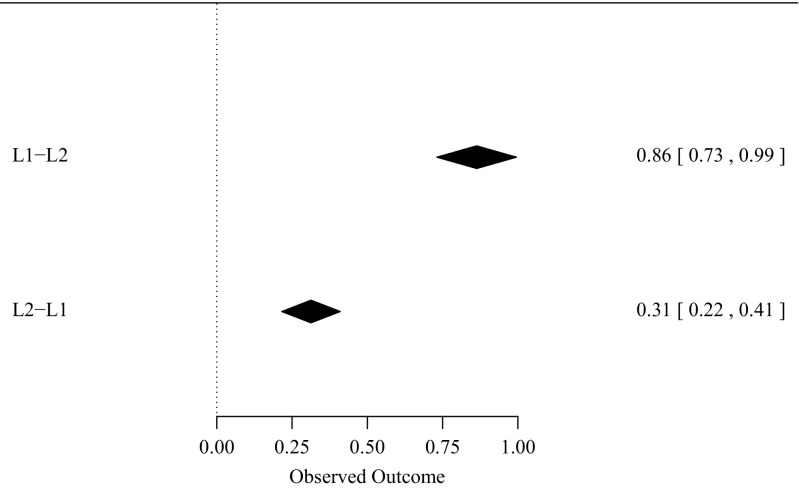
Overall effect sizes for L1–L2 and L2–L1 non-cognate masked translation priming (with their 95 % confidence intervals)

The overall effect size (d) for the L1–L2 translation priming direction was 0.86, *z* = 12.869, *p* < 0.0001, whereas the overall effect size for L2–L1 translation priming was 0.31, *z* = 6.3481, *p* < 0.0001. The difference of 0.55 between the overall effect sizes of the L1–L2 and L2–L1 translation priming directions was significant, *z* = 6.61, *p* < 0.0001. Figure 1 illustrates the effect sizes of translation priming for the two directions with 95 % CIs. Because the effect sizes are significantly different for each translation direction the next analyses were conducted for each translation direction separately.

### L1–L2 translation priming

**Fig. 2:**
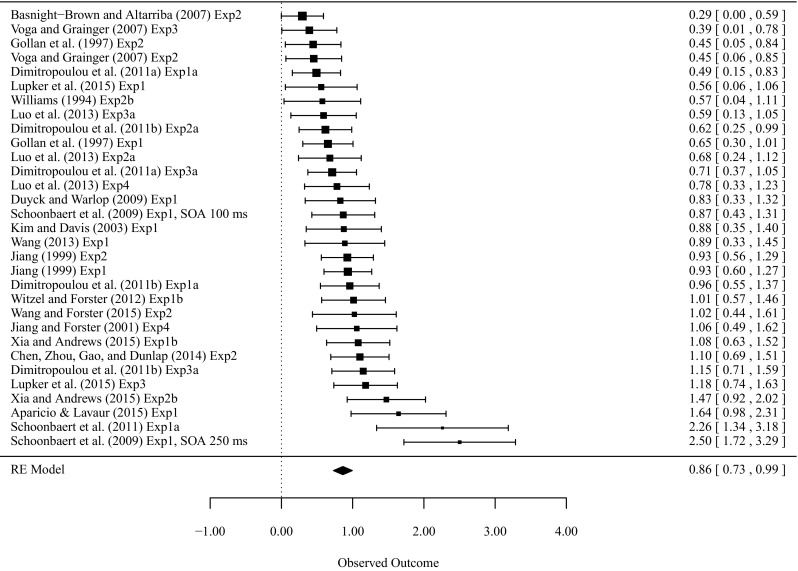
Observed effect sizes for L1–L2 non-cognate masked translation priming ordered by magnitude of the effect size and the overall effect sizes (with their 95 % confidence intervals)

Figure 2 presents an overview of the observed effect sizes for L1–L2 translation priming. A *Q* test of variance revealed that the effect sizes across experiments were heterogeneous, *Q* = 82.00, *df* = 30, *p* < 0.001. The separate random-effects model analyses that each included a different moderator revealed that ISI and SOA were the significant moderators (Table 1). Because ISI and SOA are highly correlated, *r* = 0.98, *p* < 0.001, the collinearity between ISI and SOA was reduced by using the residuals from the linear model in which ISI was predicted by SOA. When ISI and the residuals of SOA were both entered into a random-effects model, SOA was not significant anymore, β = 0.0015, SE = 0.0056, *z* = 0.262, *p* = 0.793, whereas ISI was still significant, β = 0.0022, SE = 0.0011, *z* = 2.072, *p* = 0.0382. The final model only included ISI and it explained 16.00 % of the variance between studies. The AIC (Akaike’s Information Criterion) of this model was 35.925, which is smaller than 38.177 for the model without any moderators, indicating that the model with ISI was a better model.

**Table 1:** Meta-regression analysis with one moderator for L1–L2 translation priming

	Intercept	Estimate	SE	*z* value	*p*	*R* ^2^ (%)
Number of participants	1.0951	–0.0071	0.0076	-0.942	0.346	0.00
Prime duration	0.8467	0.0004	0.0058	0.061	0.951	0.00
ISI	0.7728	0.0022	0.0010	2.128	0.033	16.00
SOA	0.6558	0.0022	0.0011	2.104	0.035	10.25
Number of items per cell	0.6905	0.0073	0.0041	1.791	0.073	4.77
Script type	0.9929	–0.1624	0.1676	–0.969	0.332	0.00
Response speed	1.7479	–0.0012	0.0008	–1.643	0.100	2.82

### L2–L1 translation priming

**Fig. 3:**
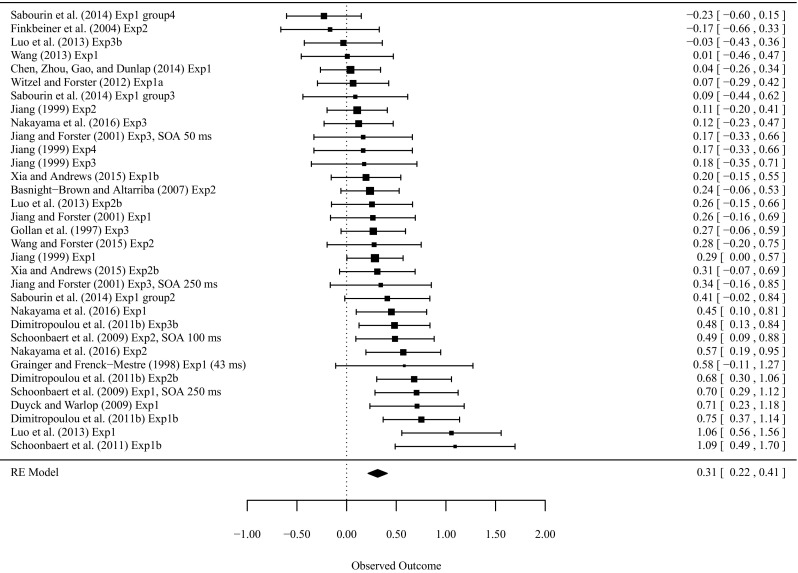
Observed effect sizes for L2–L1 masked non-cognate translation priming ordered by magnitude of the effect size and the overall effect sizes (with their 95 % confidence intervals)

Figure 3 presents an overview of the observed effect sizes for L2–L1 translation priming. A *Q* test of variance showed that the effect sizes across studies were again heterogeneous, *Q* = 60.93, *df* = 32, *p* = 0.0015. Separate random-effects models with each moderator revealed that number of items per cell was the only significant moderator (Table 2). The final model included number of items per cell as the moderator, which explained 74.60 % of the heterogeneity between studies. The AIC of this model was 7.784, which is smaller than the 17.219 for the model without any moderators, which suggested that the model with the number of items per cell was a better model.

**Table 2:** Meta-regression analysis with one moderator for L2–L1 translation priming

	Intercept	Estimate	SE	*z* value	*p*	*R* ^2^ (%)
Number of participants	0.3297	–0.0005	0.0051	–0.104	0.917	0.00
Prime duration	0.1115	0.0037	0.0040	0.935	0.350	0.00
ISI	0.3383	0.0004	0.0006	–0.619	0.536	0.00
SOA	0.3504	–0.0003	0.0006	–0.485	0.628	0.00
Number of items per cell	0.0585	0.0102	0.0026	3.914	<0.001	74.60
Script type	0.4046	–0.1214	0.1156	–1.050	0.293	0.00
Response speed	–0.1039	0.0007	0.0007	0.961	0.336	0.00

## General discussion

A meta-analysis of 64 experimental observations across 24 studies was conducted to quantitatively assess the overall effect sizes of masked translation priming effects from L1 to L2 and vice versa. The results revealed *significant* translation priming effects for *both* directions with L1–L2 translation priming significantly larger than L2–L1 translation priming (i.e., overall effects sizes: 0.86 vs. 0.31). This finding supports the view that the translation priming asymmetry between L1–L2 and L2–L1 is quantitative rather than qualitative (Schoonbaert et al., 2009).

The meta-analysis further investigated the influence of seven potential moderators (number of participants, prime duration, ISI, SOA, number of items per cell, script type and general response speed). The results revealed that the effect sizes of L1–L2 translation priming were moderated by ISI, and the effect sizes of L2–L1 translation priming were moderated by the number of items per cell.^2^ These findings have two important implications. For L1–L2 translation priming, it is very likely that a longer ISI increases the time to process the prime, resulting in a stronger priming effect. Therefore, it is crucial for future studies to systematically investigate how considerable variations in ISI influence L1–L2 translation priming. For L2–L1 translation priming, our results confirm earlier concerns in the literature about the large variation in the number of items per cell in masked translation priming experiments (Dimitropoulou et al., 2011; Nakayama et al., 2016). A possible explanation for the impact of the number of items on L2–L1 priming is that using more items may increase priming effects because it reduces the noise in the data (Van den Bussche et al., 2009). Although the number of items per cell varied from 12 to 80 in the studies included here, researchers rarely provided a justification for the choice of the number of items per cell and no studies, as far as we know, have investigated this systematically. Therefore, future studies should use a sufficient number of items per cell to investigate the L2–L1 translation priming. It is beyond the scope of the meta-analysis to provide a recommendation about the number of items per cell, but it is important to note that it is possible to calculate the number of items needed a priori for a high powered experiment (see Stevens, Mandera, Keuleers, & Brysbaert, 2015). Selecting more translation pairs could easily be accomplished by using large-scale databases with translation norms (e.g., Prior, MacWhinney, & Kroll, 2007; Tokowicz, Kroll, de Groot, & van Hell, 2002; Wen & van Heuven, 2016). Taken together, the impact of ISI and the number of experimental items should be considered when judging the mixed findings.

**Fig. 4:**
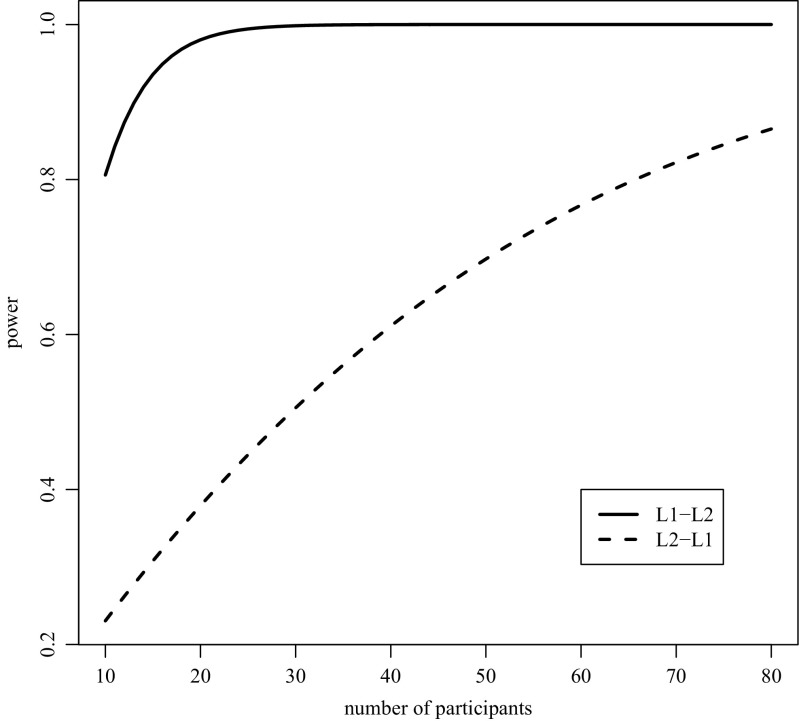
The relationship between power and number of participants estimated with the overall effect sizes for L1–L2 and L2–L1 masked non-cognate translation priming

Our meta-analysis is also useful for researchers because the overall effect sizes estimated here can be used as a benchmark to calculate the number of participants needed for a study to detect an effect. For example, in a one-tailed paired *t* test with an effect size of 0.86, only 10 participants are required to obtain a power of 0.8 for L1–L2 translation priming studies. In contrast, for a L2–L1 translation priming effect size of 0.31, 66 participants are necessary to obtain a power of 0.8 in a one-tailed repeated *t* test. As can been seen in Fig. 4, due to the differences between the effect sizes of L1–L2 and L2–L1 translation priming, there is a large difference in power between L1–L2 and L2–L1 translation priming when the same number of participants are tested.

The findings of the current meta-analysis are clear. However, there are some limitations. First of all, because the literature search did not include any unpublished data, it is not feasible to reliably estimate the publication bias in the field. Secondly, although studies have found that the L2 proficiency of bilinguals (Dimitropoulou et al., 2011; Nakayama et al., 2016) and the age of L2 acquisition (Sabourin, Brien, & Burkholder, 2014) modulated translation priming effects, we, unfortunately, could not consider the second language profile of the bilingual participants (e.g., L2 proficiency, language dominance, age of L2 exposure/acquisition) in the present study because the majority of the studies included in the present meta-analysis failed to assess or provide detailed descriptions of the participants’ second language profile. Across the 24 studies, only 12 used self-assessed proficiency ratings as an estimate of the bilinguals’ L2 proficiency, and only 10 studies provided information about the age of first L2 exposure/acquisition. Critically, studies have suggested that self-assessment is less reliable than objective language proficiency measures such as obtained with LexTALE (Khare, Verma, Kar, Srinivasan, & Brysbaert, 2013; Lemhöfer & Broersma, 2012). Surprisingly, only two studies provided detailed (objective) information about the L2 proficiency (Dimitropoulou et al., 2011; Nakayama et al., 2016). To move the field forward, future studies are strongly encouraged to include objective L2 proficiency information so that future meta-analyses can shed more light on whether or not L2 proficiency moderates translation priming.

In addition, it is crucial to notice that studies in the field seldom reported the standardized effect sizes, which is not in line with the guidelines of the American Psychological Association (2010). Future meta-analyses would benefit if standardized effect sizes of translation priming are reported (for more discussion about effect sizes, see Judd, Westfall, & Kenny, 2016; Lakens, 2013).

To summarize, we conducted the first meta-analysis of L1–L2 and L2–L1 masked translation priming in the literature, which quantitatively assessed the effect sizes of translation priming. The results not only revealed significant translation priming effects for both directions with larger L1–L2 than L2–L1 translation priming but also revealed that the effect sizes of L1–L2 were moderated by ISI and those of L2–L1 translation priming were moderated by the number of items used per cell. These findings contribute to the discussion about the mixed findings for the existing translation priming studies and provide methodological recommendations for future research.

## Electronic supplementary material

Below is the link to the electronic supplementary material.

ESM 1 (DOCX 102 kb)

## Footnotes

1 See Jiang (2015) for a comprehensive review of masked translation priming effects, including studies using other tasks (e.g., a semantic categorization task) and studies involving simultaneous bilinguals.

2 Following a reviewer’s suggestion, we also investigated the impact of script in masked experiments with only a prime duration of up to 60 ms and an ISI of up to 50 ms. The results again indicated that script type was not a significant moderator, *p*s > .80, see Supplementary Material for further details.

## References

[CR1] Altarriba J, Basnight-Brown DM (2007). Methodological considerations in performing semantic-and translation-priming experiments across languages. Behavior Research Methods.

[CR2] American Psychological Association (2010). Publication manual of the American psychological association.

[CR3] Borenstein, M., Hedges, L. V., Higgins, J. P.T., & Rothstein, H. (2009). *Introduction to Meta-Analysis.* Chichester: Wiley.

[CR4] Davis C, Sanchez-Casas R, Garcia-Albea JE, Guasch M, Molero M, Ferré P (2010). Masked translation priming: Varying language experience and word type with Spanish–English bilinguals. Bilingualism: Language and Cognition.

[CR5] de Groot AMB (1992). Determinants of word translation. Journal of Experimental Psychology: Learning, Memory, and Cognition.

[CR6] de Groot AMB, Nas GLJ (1991). Lexical representation of cognates and noncognates in compound bilinguals. Journal of Memory and Language.

[CR7] Dimitropoulou, M., Duñabeitia, J. A., & Carreiras, M. (2011). Two words, one meaning: Evidence of automatic co-activation of translation equivalents. *Frontiers in Psychology, 2*. 10.3389/fpsyg.2011.00188 PMC3155883 21886634

[CR8] Duñabeitia JA, Perea M, Carreiras M (2010). Masked translation priming effects with highly proficient simultaneous bilinguals. Experimental Psychology.

[CR9] Duyck W, Warlop N (2009). Translation priming between the native language and a second language: New evidence from Dutch-French bilinguals. Experimental Psychology.

[CR10] Finkbeiner M, Forster KI, Nicol J, Nakamura K (2004). The role of polysemy in masked semantic and translation priming. Journal of Memory and Language.

[CR11] Gollan TH, Forster KI, Frost R (1997). Translation priming with different scripts: Masked priming with cognates and noncognates in Hebrew-English bilinguals. Journal of Experimental Psychology: Learning, Memory, and Cognition.

[CR12] Hedges LV (1981). Distribution theory for Glass's estimator of effect size and related estimators. Journal of Educational and Behavioral Statistics.

[CR13] Hedges LV (1982). Estimation of effect size from a series of independent experiments. Psychological Bulletin.

[CR14] Jiang N (1999). Testing processing explanations for the asymmetry in masked cross-language priming. Bilingualism: Language and Cognition.

[CR15] Jiang N, Schwieter JW (2015). Six decades of research on bilingual presenattion. The Cambridge handbook of bilingual processing.

[CR16] Jiang N, Forster KI (2001). Cross-language priming asymmetries in lexical decision and episodic recognition. Journal of Memory and Language.

[CR17] Judd, C. M., Westfall, J., & Kenny, D. A. (2017). Experiments with more than one random factor: Designs, analytic models, and statistical power. *Annual Review of Psychology, 68*(1). 10.1146/annurev-psych-122414-033702 27687116

[CR18] Khare V, Verma A, Kar B, Srinivasan N, Brysbaert M (2013). Bilingualism and the increased attentional blink effect: Evidence that the difference between bilinguals and monolinguals generalizes to different levels of second language proficiency. Psychological Research.

[CR19] Kim J, Davis C (2003). Task effects in masked cross-script translation and phonological priming. Journal of Memory and Language.

[CR20] Kroll JF, Stewart E (1994). Category interference in translation and picture naming evidence for asymmetric connections between bilingual memory representations. Journal of Memory and Language.

[CR21] Lakens D (2013). Calculating and reporting effect sizes to facilitate cumulative science: A practical primer for t-tests and ANOVAs. Frontiers in Psychology.

[CR22] Lemhöfer K, Broersma M (2012). Introducing LexTALE: A quick and valid lexical test for advanced learners of english. Behavior Research Methods.

[CR23] Lucas M (2000). Semantic priming without association: A meta-analytic review. Psychonomic Bulletin & Review.

[CR24] Morris SB, DeShon RP (2002). Combining effect size estimates in meta-analysis with repeated measures and independent-groups designs. Psychological Methods.

[CR25] Nakayama, M., Ida, K., & Lupker, S. J. (2016). Cross-script L2-L1 noncognate translation priming in lexical decision depends on L2 proficiency: Evidence from Japanese–English bilinguals. *Bilingualism: Language and Cognition*, 1–22.

[CR26] Prior A, MacWhinney B, Kroll JF (2007). Translation norms for English and Spanish: The role of lexical variables, word class, and L2 proficiency in negotiating translation ambiguity. Behavior Research Methods.

[CR27] R Core Team. (2016). R: A Language and Environment for Statistical Computing. from http://www.R-project.org/

[CR28] Rosenthal R (1991). Meta-analytical procedures for social research.

[CR29] Sabourin L, Brien C, Burkholder M (2014). The effect of age of L2 acquisition on the organization of the bilingual lexicon: Evidence from masked priming. Bilingualism: Language and Cognition.

[CR30] Schoonbaert S, Duyck W, Brysbaert M, Hartsuiker RJ (2009). Semantic and translation priming from a first language to a second and back: Making sense of the findings. Memory & Cognition.

[CR31] Siyanova-Chanturia A, Conklin K, van Heuven WJB (2011). Seeing a phrase “time and again” matters: The role of phrasal frequency in the processing of multiword sequences. Journal of Experimental Psychology: Learning, Memory, and Cognition.

[CR32] Stevens, M., Mandera, P., Keuleers, E., & Brysbaert, M. (2015). *How spurious can you get? Charting the sensitivity and specificity of typical psycholinguistic experiments with megastudy data.* Paper presented at the 19th Conference of the European Society for Cognitive Psychology.

[CR33] Tokowicz N, Kroll JF, de Groot AMB, van Hell JG (2002). Number-of-translation norms for Dutch-English translation pairs: A new tool for examining language production. Behavior Research Methods, Instruments, & Computers.

[CR34] Van den Bussche E, Van den Noortgate W, Reynvoet B (2009). Mechanisms of masked priming: A meta-analysis. Psychological Bulletin.

[CR35] Viechtbauer W (2010). Conducting meta-analyses in R with the metafor package. Journal of Statistical Software.

[CR36] Wen, Y., & van Heuven, W. J. B. (2016). Chinese translation norms for 1429 English words. *Behavior Research Methods*. doi:10.3758/s13428-016-0761-x. 10.3758/s13428-016-0761-x PMC5429370 27325164

[CR37] Witzel NO, Forster KI (2012). How L2 words are stored: The episodic L2 hypothesis. Journal of Experimental Psychology: Learning, Memory, and Cognition.

[CR38] Xia V, Andrews S (2015). Masked translation priming asymmetry in Chinese-English bilinguals: Making sense of the Sense Model. The Quarterly Journal of Experimental Psychology.

