# Supplementary material for: Non-cognate translation priming in masked priming lexical decision experiments: A meta-analysis
Source: Psychon Bull Rev. 2016 Sep 9;24(3):879–86. doi: 10.3758/s13423-016-1151-1 (PMC5486879; doi:10.3758/s13423-016-1151-1)
Supplement: Supplementary file 1 — (DOCX 102 kb) [file 13423_2016_1151_MOESM1_ESM.docx]

**Supplementary Material**

**Non-cognate translation priming in masked priming lexical decision experiments: A meta-analysis**

Table S1 Description of 31 experiments extracted from 20 studies using non-cognate masked L1-L2 translation priming paradigm with a lexical decision task. The average L1-L2 translation priming effect was 44 ms (range: 12 -127 ms). **NoP**: number of participants; **NoI**: number of items per cell; **RT-c**: reaction times in the control conditions; **PE**: priming effects.

| **Study** | **Experiment** | **L1** | **L2** | **Prime**  **(ms)** | **ISI**  **(ms)** | **SOA**  **(ms)** | **NoP** | **NoI** | **RT_c**  **(ms)** | **PE**  **(ms)** |
| --- | --- | --- | --- | --- | --- | --- | --- | --- | --- | --- |
| [Williams (1994](#_ENREF_22)) | Exp2b | German/Italian/French | English | 40 | 10 | 50 | 18 | 9 | 723 | 33* |
| [Gollan et al. (1997](#_ENREF_8)) | Exp1 | Hebrew | English | 50 | 0 | 50 | 40 | 16 | 712 | 36*** |
|  | Exp2 | English | Hebrew | 50 | 0 | 50 | 30 | 16 | 979 | 52* |
| [Jiang (1999](#_ENREF_10)) | Exp1 | Chinese | English | 50 | 0 | 50 | 52 | 16 | 755 | 45* |
|  | Exp2 | Chinese | English | 50 | 0 | 50 | 44 | 16 | 814 | 68* |
| [Jiang and Forster (2001](#_ENREF_11)) | Exp4 | Chinese | English | 50 | 0 | 50 | 22 | 16 | 717 | 41* |
| [Kim and Davis (2003](#_ENREF_12)) | Exp1 | Korean | English | 50 | 0 | 50 | 22 | 12 | 674 | 40* |
| [Basnight-Brown and Altarriba (2007](#_ENREF_2)) | Exp2 | Spanish | English | 100 | 0 | 100 | 48 | 16 | 636 | 33*** |
| [Voga and Grainger (2007](#_ENREF_19)) | Exp2 | Greek | French | 50 | 0 | 50 | 30 | 15 | 684 | 23* |
|  | Exp3 | Greek | French | 50 | 0 | 50 | 30 | 15 | 691 | 22* |
| [Duyck and Warlop (2009](#_ENREF_6)) | Exp1 | Dutch | French | 56 | 56 | 112 | 24 | 22 | 687 | 48*** |
| [Schoonbaert et al. (2009](#_ENREF_17)) | Exp1 | Dutch | English | 50 | 50 | 100 | 30 | 28 | 572 | 19*** |
|  | Exp1 | Dutch | English | 50 | 200 | 250 | 30 | 28 | 661 | 100*** |

Table S1 (*Continued*)

| **Study** | **Experiment** | **L1** | **L2** | **Prime**  **(ms)** | **ISI**  **(ms)** | **SOA**  **(ms)** | **NoP** | **NoI** | **RT_c**  **(ms)** | **PE**  **(ms)** |
| --- | --- | --- | --- | --- | --- | --- | --- | --- | --- | --- |
| [Dimitropoulou, Duñabeitia, and Carreiras (2011a](#_ENREF_4)) | Exp1a | Greek | Spanish | 50 | 0 | 50 | 40 | 14 | 777 | 29** |
|  | Exp3a | Greek | Spanish | 50 | 50 | 100 | 44 | 14 | 818 | 31*** |
| [Dimitropoulou, Duñabeitia, and Carreiras (2011b](#_ENREF_5)) | Exp1a | Greek | English | 50 | 0 | 50 | 36 | 58 | 723 | 31*** |
|  | Exp2a | Greek | English | 50 | 0 | 50 | 36 | 58 | 739 | 28*** |
|  | Exp3a | Greek | English | 50 | 0 | 50 | 36 | 58 | 695 | 28*** |
| [Schoonbaert, Holcomb, Grainger, and Hartsuiker (2011](#_ENREF_18)) | Exp1a | English | French | 100 | 20 | 120 | 20 | 80 | 653 | 70*** |
| [Witzel and Forster (2012](#_ENREF_23)) | Exp1b | Chinese | English | 50 | 0 | 50 | 32 | 16 | 678 | 39*** |
| [Luo et al. (2013](#_ENREF_13)) | Exp2a | Chinese | English | 50 | 150 | 200 | 27 | 12 | 745 | 77** |
|  | Exp3a | Chinese | English | 50 | 150 | 200 | 24 | 12 | 803 | 75** |
|  | Exp4 | Chinese | English | 50 | 150 | 200 | 27 | 12 | 598 | 30*** |
| [Wang (2013](#_ENREF_20)) | Exp1 | English | Chinese | 50 | 0 | 50 | 20 | 40 | 604 | 39** |
| [Chen et al. (2014](#_ENREF_3)) | Exp2 | Chinese | English | 50 | 150 | 200 | 40 | 14 | 678 | 32*** |
| [Aparicio and Lavaur (2015](#_ENREF_1)) | Exp1 | French | English | 50 | 17 | 67 | 24 | 20 | 574 | 14** |

Table S1 (*Continued*)

| **Study** | **Experiment** | **L1** | **L2** | **Prime**  **(ms)** | **ISI**  **(ms)** | **SOA**  **(ms)** | **NoP** | **NoI** | **RT_c**  **(ms)** | **PE**  **(ms)** |
| --- | --- | --- | --- | --- | --- | --- | --- | --- | --- | --- |
| [Lupker, Perea, and Nakayama (2015](#_ENREF_14)) | Exp1 | Spanish | English | 50 | 0 | 50 | 20 | 50 | 620 | 12* |
|  | Exp3 | Japanese | English | 60 | 0 | 60 | 36 | 30 | 668 | 45*** |
| [Wang and Forster (2015](#_ENREF_21)) | Exp2 | Chinese | English | 50 | 0 | 50 | 20 | 15 | 625 | 32* |
| [Xia and Andrews (2015](#_ENREF_24)) | Exp1b | Chinese | English | 50 | 150 | 200 | 34 | 16 | 854 | 127*** |
|  | Exp2b | Chinese | English | 50 | 150 | 200 | 30 | 16 | 647 | 76*** |

*Note.* * *p* < 0.05, ** *p* < 0.01, *** *p* < 0.001

Table S2 Description of 33 experiments extracted from 18 studies using non-cognate masked L2-L1 translation priming paradigm with a lexical decision task. The average L2-L1 translation priming effect was 11 ms (range: -4 - 48 ms) and 13 of the 33 experiments (39.4%) reported significant effects. **NoP**: number of participants; **NoI**: number of items per cell; **RT-c**: reaction times in the control conditions; **PE**: priming effects

| **Study** | **Experiment** | **L1** | **L2** | **Prime**  **(ms)** | **ISI**  **(ms)** | **SOA**  **(ms)** | **NoP** | **NoI** | **RT_c**  **(ms)** | **PE**  **(ms)** |
| --- | --- | --- | --- | --- | --- | --- | --- | --- | --- | --- |
| [Gollan, Forster, and Frost (1997](#_ENREF_8)) | Exp3 | Hebrew | English | 50 | 0 | 50 | 40 | 16 | 574 | 9 |
| [Grainger and Frenck-Mestre (1998](#_ENREF_9)) | Exp1 (43 ms) | English | French | 43 | 14 | 57 | 12 | 30 | 573 | 10 |
| [Jiang (1999](#_ENREF_10)) | Exp1 | Chinese | English | 50 | 0 | 50 | 52 | 16 | 581 | 13* |
|  | Exp2 | Chinese | English | 50 | 50 | 50 | 44 | 16 | 635 | 3 |
|  | Exp3 | Chinese | English | 50 | 50 | 100 | 16 | 16 | 583 | 4 |
|  | Exp4 | Chinese | English | 50 | 200 | 250 | 18 | 16 | 541 | 7 |
| [Jiang and Forster (2001](#_ENREF_11)) | Exp1 | Chinese | English | 50 | 200 | 250 | 24 | 16 | 580 | 8 |
|  | Exp3, SOA 250 ms | Chinese | English | 50 | 200 | 250 | 18 | 16 | 552 | 9 |
|  | Exp3, SOA 50 ms | Chinese | English | 50 | 0 | 50 | 18 | 16 | 522 | 4 |
| [Finkbeiner, Forster, Nicol, and Nakamura (2004](#_ENREF_7)) | Exp2 | Japanese | English | 50 | 150 | 200 | 18 | 26 | 525 | -4 |

Table S2 (*Continued*)

| **Study** | **Experiment** | **L1** | **L2** | **Prime**  **(ms)** | **ISI**  **(ms)** | **SOA**  **(ms)** | **NoP** | **NoI** | **RT_c**  **(ms)** | **PE**  **(ms)** |
| --- | --- | --- | --- | --- | --- | --- | --- | --- | --- | --- |
| [Basnight-Brown and Altarriba (2007](#_ENREF_2)) | Exp2 | Spanish | English | 100 | 0 | 100 | 48 | 16 | 728 | 24** |
| [Duyck and Warlop (2009](#_ENREF_6)) | Exp1 | Dutch | French | 56 | 56 | 112 | 24 | 22 | 544 | 26** |
| [Schoonbaert, Duyck, Brysbaert, and Hartsuiker (2009](#_ENREF_17)) | Exp2 | Dutch | English | 50 | 50 | 100 | 30 | 28 | 506 | 12** |
|  | Exp2 | Dutch | English | 50 | 200 | 250 | 30 | 28 | 554 | 28*** |
| [Dimitropoulou et al. (2011b](#_ENREF_5)) | Exp1b | Greek | English | 50 | 0 | 50 | 36 | 58 | 684 | 14*** |
|  | Exp2b | Greek | English | 50 | 0 | 50 | 36 | 58 | 656 | 14*** |
|  | Exp3b | Greek | English | 50 | 0 | 50 | 36 | 58 | 643 | 11** |
| [Schoonbaert et al. (2011](#_ENREF_18)) | Exp1b | English | French | 100 | 20 | 120 | 20 | 80 | 583 | 24*** |
| [Witzel and Forster (2012](#_ENREF_23)) | Exp1a | Chinese | English | 50 | 200 | 250 | 32 | 16 | 525 | 1 |
| [Luo et al. (2013](#_ENREF_13)) | Exp1 | Chinese | English | 50 | 150 | 200 | 27 | 12 | 673 | 48** |
|  | Exp2b | Chinese | English | 50 | 150 | 200 | 26 | 12 | 659 | 30 |
|  | Exp3b | Chinese | English | 50 | 150 | 200 | 27 | 12 | 753 | -4 |
| [Wang (2013](#_ENREF_20)) | Exp1 | English | Chinese | 50 | 0 | 50 | 20 | 40 | 483 | 0 |

Table S2 (*Continued*)

| **Study** | **Experiment** | **L1** | | **L2** | **Prime**  **(ms)** | **ISI**  **(ms)** | **SOA**  **(ms)** | **NoP** | **NoI** | **RT_c**  **(ms)** | | **PE**  **(ms)** |
| --- | --- | --- | --- | --- | --- | --- | --- | --- | --- | --- | --- | --- |
| [Chen, Zhou, Gao, and Dunlap (2014](#_ENREF_3)) | Exp1 | Chinese | English | | 50 | 150 | 200 | 44 | 14 | 555 | 4 | |
| [Sabourin, Brien, and Burkholder (2014](#_ENREF_16)) | Exp1 group2 | English | French | | 50 | 0 | 50 | 25 | 15.5 | <550,>525 | 14.2* | |
|  | Exp1 group3 | English | French | | 50 | 0 | 50 | 16 | 15.5 | <550,>525 | 0.8 | |
|  | Exp1 group4 | English | French | | 50 | 0 | 50 | 30 | 15.5 | about 550 | -0.4 | |
| [Wang and Forster (2015](#_ENREF_21)) | Exp2 | Chinese | English | | 80 | 0 | 80 | 20 | 15 | 530 | 5 | |
| [Xia and Andrews (2015](#_ENREF_24)) | Exp1b | Chinese | English | | 50 | 150 | 200 | 34 | 16 | 620 | 12 | |
|  | Exp2b | Chinese | English | | 50 | 150 | 200 | 30 | 16 | 591 | 14 | |
| [Nakayama, Ida, and Lupker (2016](#_ENREF_15)) | Exp1 | Japanese | English | | 50 | 0 | 50 | 36 | 30 | 518 | 10* | |
|  | Exp2 | Japanese | English | | 60 | 0 | 60 | 34 | 30 | 533 | 22** | |
|  | Exp3 | Japanese | English | | 60 | 0 | 60 | 34 | 30 | 509 | 3 | |

*Note.* * *p* < 0.05, ** *p* < 0.01, *** *p* < 0.001

We also explored the impact of script in a subset of masked experiments with a prime duration of up to 60 ms and an ISI of up to 50 ms. This analysis included 23 experiments in L1-L2 direction (17 different-script studies and 6 same-script studies) and 20 experiments in L2-L1 direction (13 different-script studies and 7 same-script studies). For both directions, script type was not a significant moderator, *p*s > .80. The results of random-effects model conducted to estimate the effect sizes of L1-L2 and L2-L1 translation priming in same and different scripts are shown in Figure S1.

Figure S1 Overall effect sizes for L1-L2 and L2-L1 translation priming involving different script and same script in healily masked experiements (with their 95% confidence intervals)

**References**

Aparicio, X., & Lavaur, J.-M. (2015). Masked Translation Priming Effects in Visual Word Recognition by Trilinguals. *Journal of Psycholinguistic Research*, 1-20.

Basnight-Brown, D. M., & Altarriba, J. (2007). Differences in semantic and translation priming across languages: The role of language direction and language dominance. *Memory & Cognition, 35*(5), 953-965.

Chen, B., Zhou, H., Gao, Y., & Dunlap, S. (2014). Cross-language translation priming asymmetry with Chinese-English bilinguals: A test of the sense model. *Journal of Psycholinguistic Research, 43*(3), 225-240.

Dimitropoulou, M., Duñabeitia, J. A., & Carreiras, M. (2011a). Masked translation priming effects with low proficient bilinguals. *Memory & Cognition, 39*(2), 260-275.

Dimitropoulou, M., Duñabeitia, J. A., & Carreiras, M. (2011b). Two words, one meaning: Evidence of automatic co-activation of translation equivalents. *Frontiers in Psychology, 2*.

Duyck, W., & Warlop, N. (2009). Translation priming between the native language and a second language: New evidence from Dutch-French bilinguals. *Experimental Psychology, 56*(3), 173-179.

Finkbeiner, M., Forster, K. I., Nicol, J., & Nakamura, K. (2004). The role of polysemy in masked semantic and translation priming. *Journal of Memory and Language, 51*(1), 1-22.

Gollan, T. H., Forster, K. I., & Frost, R. (1997). Translation priming with different scripts: Masked priming with cognates and noncognates in Hebrew-English bilinguals. *Journal of Experimental Psychology: Learning, Memory, and Cognition, 23*(5), 1122-1139.

Grainger, J., & Frenck-Mestre, C. (1998). Masked priming by translation equivalents in proficient bilinguals. *Language and Cognitive Processes, 13*(6), 601-623.

Jiang, N. (1999). Testing processing explanations for the asymmetry in masked cross-language priming. *Bilingualism: Language and Cognition, 2*(1), 59-75.

Jiang, N., & Forster, K. I. (2001). Cross-language priming asymmetries in lexical decision and episodic recognition. *Journal of Memory and Language, 44*(1), 32-51.

Kim, J., & Davis, C. (2003). Task effects in masked cross-script translation and phonological priming. *Journal of Memory and Language, 49*(4), 484-499.

Luo, X., Cheung, H., Bel, D., Li, Li, Chen, L., & Mo, L. (2013). The roles of semantic sense and form-meaning connection in translation priming. *The Psychological Record, 63*(1), 193.

Lupker, S. J., Perea, M., & Nakayama, M. (2015). Non-cognate translation priming effects in the same-different task: Evidence for the impact of “higher level” information. *Language, Cognition and Neuroscience, 30*(7), 781-795.

Nakayama, M., Ida, K., & Lupker, S. J. (2016). Cross-script L2-L1 noncognate translation priming in lexical decision depends on L2 proficiency: Evidence from Japanese–English bilinguals. *Bilingualism: Language and Cognition*, 1-22.

Sabourin, L., Brien, C., & Burkholder, M. (2014). The effect of age of L2 acquisition on the organization of the bilingual lexicon: Evidence from masked priming. *Bilingualism: Language and Cognition, 17*(3), 542-555.

Schoonbaert, S., Duyck, W., Brysbaert, M., & Hartsuiker, R. J. (2009). Semantic and translation priming from a first language to a second and back: Making sense of the findings. *Memory & Cognition, 37*(5), 569-586.

Schoonbaert, S., Holcomb, P. J., Grainger, J., & Hartsuiker, R. J. (2011). Testing asymmetries in noncognate translation priming: Evidence from RTs and ERPs. *Psychophysiology, 48*(1), 74-81.

Voga, M., & Grainger, J. (2007). Cognate status and cross-script translation priming. *Memory & Cognition 35*(5), 938-952.

Wang, X. (2013). Language dominance in translation priming: Evidence from balanced and unbalanced Chinese–English bilinguals. *The Quarterly Journal of Experimental Psychology, 66*(4), 727-743.

Wang, X., & Forster, K. I. (2015). Is translation priming asymmetry due to partial awareness of the prime? *Bilingualism: Language and Cognition, 18*(4), 651-669.

Williams, J. N. (1994). The relationship between word meanings in the first and second language: Evidence for a common, but restricted, semantic code. *European Journal of Cognitive Psychology, 6*(2), 195-220.

Witzel, N. O., & Forster, K. I. (2012). How L2 words are stored: The episodic L2 hypothesis. *Journal of Experimental Psychology: Learning, Memory, and Cognition, 38*(6), 1608-1621.

Xia, V., & Andrews, S. (2015). Masked translation priming asymmetry in Chinese-English bilinguals: Making sense of the Sense Model. *The Quarterly Journal of Experimental Psychology, 68*(2), 294-325.
